# Chloroplast genome sequence of Chongming lima bean (*Phaseolus lunatus* L.) and comparative analyses with other legume chloroplast genomes

**DOI:** 10.1186/s12864-021-07467-8

**Published:** 2021-03-18

**Authors:** Shoubo Tian, Panling Lu, Zhaohui Zhang, Jian Qiang Wu, Hui Zhang, Haibin Shen

**Affiliations:** 1 Shanghai Key Laboratory of Protected Horticultural Technology, Horticultural Research Institute, Shanghai Academy of Agricultural Sciences, Shanghai, 201403 China; 2 Nanjing Agricultural University, NanJing, 210095 China

**Keywords:** *Phaseolus lunatus*, Chloroplast genome, Leguminosae, Phylogenetic relationship, Comparative analysis

## Abstract

**Background:**

Lima bean (*Phaseolus lunatus* L.) is a member of subfamily Phaseolinae belonging to the family Leguminosae and an important source of plant proteins for the human diet. As we all know, lima beans have important economic value and great diversity. However, our knowledge of the chloroplast genome level of lima beans is limited.

**Results:**

The chloroplast genome of lima bean was obtained by Illumina sequencing technology for the first time. The Cp genome with a length of 150,902 bp, including a pair of inverted repeats (IRA and IRB 26543 bp each), a large single-copy (LSC 80218 bp) and a small single-copy region (SSC 17598 bp). In total, 124 unique genes including 82 protein-coding genes, 34 tRNA genes, and 8 rRNA genes were identified in the *P. lunatus* Cp genome. A total of 61 long repeats and 290 SSRs were detected in the lima bean Cp genome. It has a typical 50 kb inversion of the Leguminosae family and an 70 kb inversion to subtribe Phaseolinae. *rpl16*, *accD*, *petB*, *rsp16*, *clpP*, *ndhA*, *ndhF* and *ycf1* genes in coding regions was found significant variation, the intergenic regions of *trnk*-*rbcL*, *rbcL*-*atpB*, *ndhJ*-*rps4*, *psbD*-*rpoB*, *atpI*-*atpA*, *atpA*-*accD*, *accD*-*psbJ*, *psbE*-*psbB*, *rsp11*-*rsp19*, *ndhF*-*ccsA* was found in a high degree of divergence. A phylogenetic analysis showed that *P. lunatus* appears to be more closely related to *P. vulgaris*, *V.unguiculata* and *V. radiata*.

**Conclusions:**

The characteristics of the lima bean Cp genome was identified for the first time, these results will provide useful insights for species identification, evolutionary studies and molecular biology research.

**Supplementary Information:**

The online version contains supplementary material available at 10.1186/s12864-021-07467-8.

## Background

Lima bean (*Phaseolus lunatus* L.) is one of five species domesticated within *Phaseolus*, together with common bean (*P. vulgaris* L.), scarlet runner bean (*P. coccineus* L.), tepary bean (*P. acutifolius* A. Gray) and year bean (*P. polyanthus* Greenm) [1]. Lima beans play an important role in the human diet as an important source of protein when common beans do not grow well in warmer and drier regions [2]. Wild lima bean have three gene pools, two Mesoamerican pools (MI and MII) and the Andean pool (AI) [3]. Lima bean is a self-compatible annual or short living perennial and predominantly self-pollinating species with a mixed-mating system, it was used as a plant model due to its alternating outbreeder-inbreederbehavior [4, 5]. The cultivated form is widely distributed all over the world, Chongming lima bean, an important characteristic vegetable variety in the Chongming area, has been grown on Chongming Island for more than 100 years [6].

Chloroplasts, a place for plant photosynthesis, starch, fatty acids and amino acids biosynthesis, play an important role in the transfer and expression of genetic material [7]. Chloroplast has its own genome, chloroplast genome of most plants are mostly double-stranded circular, but a few species have linear forms with multiple copies. The genome size usually ranges from120 to 170 kb and includes 120–130 genes [8]. It has a typical quarter structure, which composed of a large single-copy region, a small single-copy region and a pair of large inverted repeats [9–11]. The Cp genome is highly conserved, the differences between different plant species are mainly caused by the IR region’s contraction and expansion [12, 13]. With the development of high-throughput sequencing technologies, there were more than 2400 plant Cp genomes have been published in the NCBI database [14]. Leguminosae, with nearly 770 genera and more than 19,500 species, is the third largest family of angiosperms [15]. Within the Leguminosae family, there were more than 44 species Cp genomes have been published including *C. arietinum* [8], *G. gracilis* [16], *L. japonica* [17], *C. tetragonoloba* [18], *G. max* [19], *V. radiate* [20], and *P. vulgaris* [21]. Leguminosae has experienced a great number of plastid genomic rearrangements [22], including loss of one copy of the IR [23, 24], inversion of 50 kb and 70 kb [17, 21, 25], transfer of *infA*, *rpl22* and *accD* genes to the nucleus [26–28] and loss of the *rps12* and *clpP* introns [8, 26].

Chloroplast DNA has been extensively used to taxonomy, phylogenetics and evolution of plants, due to its low substitution rates of nucleotide and relatively conserved structural variation of genomic [29–31]. Phylogenetic analyses of Leguminosae were mainly based on gene fragments in chloroplast DNA like *trnL*, rbcL and *matK* [32–34]. Based on the chloroplast *matk* gene and combining the characteristics of morphology, chemistry and chromosome number, a new classification system of six subfamilies was proposed, and the most complete leguminous phylogeny tree was constructed so far [15]. However, the classification and phylogenetic relationships of the main branches within the subfamilies are still unclear. Chloroplast phylogenetic genome has been successfully used to analyze the phylogenetic relationship of many difficult groups, and it also provided a better system framework for studying the structural characteristics, variation and evolution of plants [35, 36]. Due to the limited chloroplast genomes of legumes that have been sequenced, phylogenetic chloroplast phylogeny has not been applied to classification of the Leguminosae.

Currently, there are no published studies of the Cp genome of lima bean. In this study, we applied a combination of de novo and reference-guides to assemble complete Cp genome sequence of *P. lunatus*. Here, we not only described the whole Cp genome sequence of *P. lunatus* and the characteristics of long repeats and SSRs, but also compared and analysed the Cp genome with other members of Leguminosae. It is expected that the results will help us to understand of the Cp genome of lima bean and provide markers for phylogenetic and genetic studies.

## Results

### Characteristics of the *P. lunatus* L. Cp genome

**Fig. 1:**
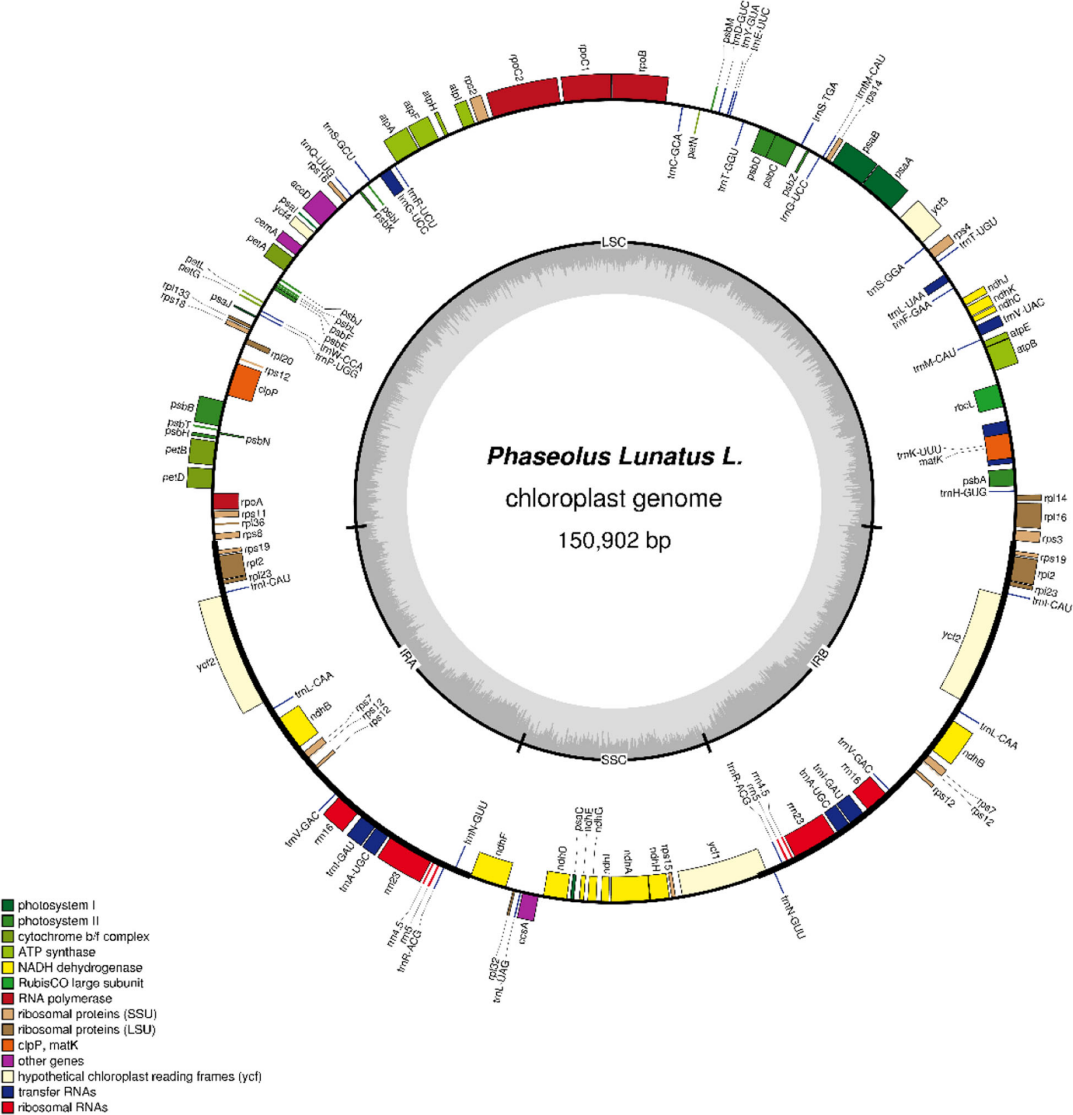
Gene map of the *P.lunatus.* Chloroplast genome

The Cp genome of lima bean was 150,902 bp in size with a typical quadripartite structure, containing a pair of inverted repeats (IRs; 26,543 bp), a large single copy (LSC; 80,218 bp) and a small single copy (SSC; 17,598 bp) (Fig. 1). The GC content in lima bean was 35.44%, the GC content of LSC, SSC and IR regions was 32.92, 28.61 and 41.52% respectively (Table 1), IR regions was higher than the LSC and SSC regions. Species of Leguminous: *G. max*, *P. vulgaris*, *V. unguiculata*, *G. sojasieb*, *V. faba* and *P. sativum* were selected to Compare with lima bean (Table 2). Although the sizes of the overall genome had differences, the GC content was similar in each region (LSC, SSC and IR) of different species. There is a litter difference in total genes, CDS and tRNAs among the seven species. *C. cajan* has most genes, CDS and tRNAs and *V. radiata* has least.

**Table 1:** Base composition of the *P.lunatus*. Chloroplast genome

Region	A(%)	C(%)	G(%)	T(U)(%)	A + T(%)	G + C(%)
LSC	33.87	15.97	16.95	33.22	67.09	32.92
SSC	35.36	15.19	13.42	36.03	71.39	28.61
IRa	29.47	21.55	19.98	29.01	58.48	41.53
IRb	29.01	19.98	21.55	29.47	58.48	41.53
Total	32.41	17.56	17.88	32.15	64.56	35.44

**Table 2:** Comparison analyses of Cp genomes among six Leguminosae species

Species	Genome size (bp)	LSC (bp)	SSC (bp)	IR (bp)	Number of genes	Protein-coding genes (CDS)	tRNA genes	rRNA genes	GC content(%)
*C. cajan*	152,242	83,369	17,815	25,529	134	87	39	8	34.97
*G. max*	152,218	83,175	17,895	25,574	128	83	37	8	35.37
*G. soja*	152,217	83,174	17,895	25,574	129	83	38	8	35.38
*P. lunatus.*	150,902	80,218	17,598	26,543	127	82	37	8	35.44
*P. vulgaris*	150,285	79,823	17,610	26,426	127	83	36	8	35.44
*V. radiata*	151,271	80,898	17,425	26,474	126	82	36	8	35.23
*V. unguiculata*	152,415	81,822	17,425	26,584	130	84	38	8	35.24

There were 129 genes found in the *P. lunatus* Cp genome, containing 82 protein-coding genes, 37 tRNA genes, 8 rRNA genes and 2 pseudogenes (Tables 2 and 3). There are 79 genes (56 protein-coding and 23 tRNAs) located in LSC region and 13 genes (12 CDS and 1tRNA) in SSC region. Among them, 35 genes (13 CDS, 14 tRNAs and 8 rRNAs genes) were duplicated in the IR regions (Fig. 1; Table S1). Codon usage frequency of the *P. lunatus* Cp genome was estimated and summarized (Table S2). Totally, all the genes are encoded by 25,873 codons, in these codons, the most frequent amino acids are leucine (2719, 10.51%) and the least are cysteine (300, 1.16%). The most preferred synonymous codons end with A and U.

**Table 3:** The genes present in the *P.lunatus*

Category	Gene group	Gene name
Photosynthesis	Subunits of photosystem I	*psaA, psaB, psaC, psaI, psaJ*
	Subunits of photosystem II	*psbA, psbB, psbC, psbD, psbE, psbF, psbH, psbI, psbJ, psbK, psbL, psbM, psbN, psbT, psbZ*
	Subunits of NADH dehydrogenase	ndhA*, ndhB* (2), ndhC, ndhD, ndhE, ndhF, ndhG, ndhH, ndhI, ndhJ, ndhK
	Subunits of cytochrome b/f complex	petA, petB*, petD*, petG, petL, petN
	Subunits of ATP synthase	atpA, atpB, atpE, atpF*, atpH, atpI
	Large subunit of rubisco	*rbcL*
Self-replication	Proteins of large ribosomal subunit	#rpl133, rpl14, rpl16*, rpl2* (2), rpl20, rpl23 (2), rpl32, rpl36
	Proteins of small ribosomal subunit	#rps16, rps11, rps12**(2), rps14, rps15, rps18, rps19 (2), rps2, rps3, rps4, rps7 (2), rps8
	Subunits of RNA polymerase	rpoA, rpoB, rpoC1*, rpoC2
	Ribosomal RNAs	*rrn16 (2), rrn23 (20, rrn4.5 (2), rrn5 (2)*
	Transfer RNAs	trnA-UGC*(2),trnC-GCA,trnD-GUC,trnE-UUC,trnF-GAA,trnG-UCC,trnG-UCC*,trnH-GUG,trnI-CAU (2),trnI-GAU* (2),trnK-UUU*,trnL-CAA (2),trnL-UAA*,trnL-UAG,trnM-CAU,trnN-GUU(2),trnP-UGG,trnQ-UUG,trnR-ACG (2),trnR-UCU,trnS-GCU,trnS-GGA,trnS-TGA,trnT-GGU,trnT-UGU,trnV-GAC (2),trnV-UAC*,trnW-CCA,trnY-GUA,trnfM-CAU
Other genes	Maturase	*matK*
	Protease	clpP**
	Envelope membrane protein	*cemA*
	Acetyl-CoA carboxylase	*accD*
	c-type cytochrome synthesis gene	*ccsA*
Genes of unknown function	Conserved hypothetical chloroplast ORF	ycf1, ycf2 (2), ycf3**, ycf4

Notes: Gene*: Gene with one intron; Gene**: Gene with two introns; #Gene: Pseudo gene; Gene (2): Number of copies of multi-copy genes;

Overall, 22 intron-containing genes (14 protein-coding genes and 8 tRNA genes) were found (Table 4). Among them, 20 genes have one intron, *ycf3* and *clpP* have two introns. *trnL-UAA* and *trnK-UUU* have the the smallest intron (467 bp) and largest intron (2562 bp), respectively. In the *P. lunatus* Cp genome, *rps16* and *rpl133* gene was found to be present as a pseudogene.

**Table 4:** The lengths of exons and introns in genes with introns in the *P. lunatus.* Chloroplast genome

Gene	Location	Exon I (bp)	Intron I (bp)	Exon II (bp)	Intron II (bp)	Exon III (bp)
*rpl16*	LSC	9	1036	402		
*trnK-UUU*	LSC	37	2562	35		
*trnV-UAC*	LSC	38	576	37		
*trnL-UAA*	LSC	37	467	50		
*ycf3*	LSC	129	683	228	797	153
*rpoC1*	LSC	435	812	1620		
*atpF*	LSC	144	730	399		
*trnG-UCC*	LSC	23	694	49		
*rps12*	IRa	114	–	231	534	24
*clpP*	LSC	68	741	297	716	223
*petB*	LSC	6	792	642		
*petD*	LSC	9	717	474		
*rpl2*	IRb	393	616	471		
*ndhB*	IRb	723	691	756		
*rps12*	IRb	231	–	24	534	114
*trnI-GAU*	IRb	42	935	35		
*trnA-UGC*	IRb	38	811	35		
*ndhA*	SSC	552	1292	540		
*trnA-UGC*	IRa	38	811	35		
*trnI-GAU*	IRa	42	935	35		
*ndhB*	IRa	723	691	756		
*rpl2*	IRa	393	616	471		

### Long repeats and SSRs

**Fig. 2:**
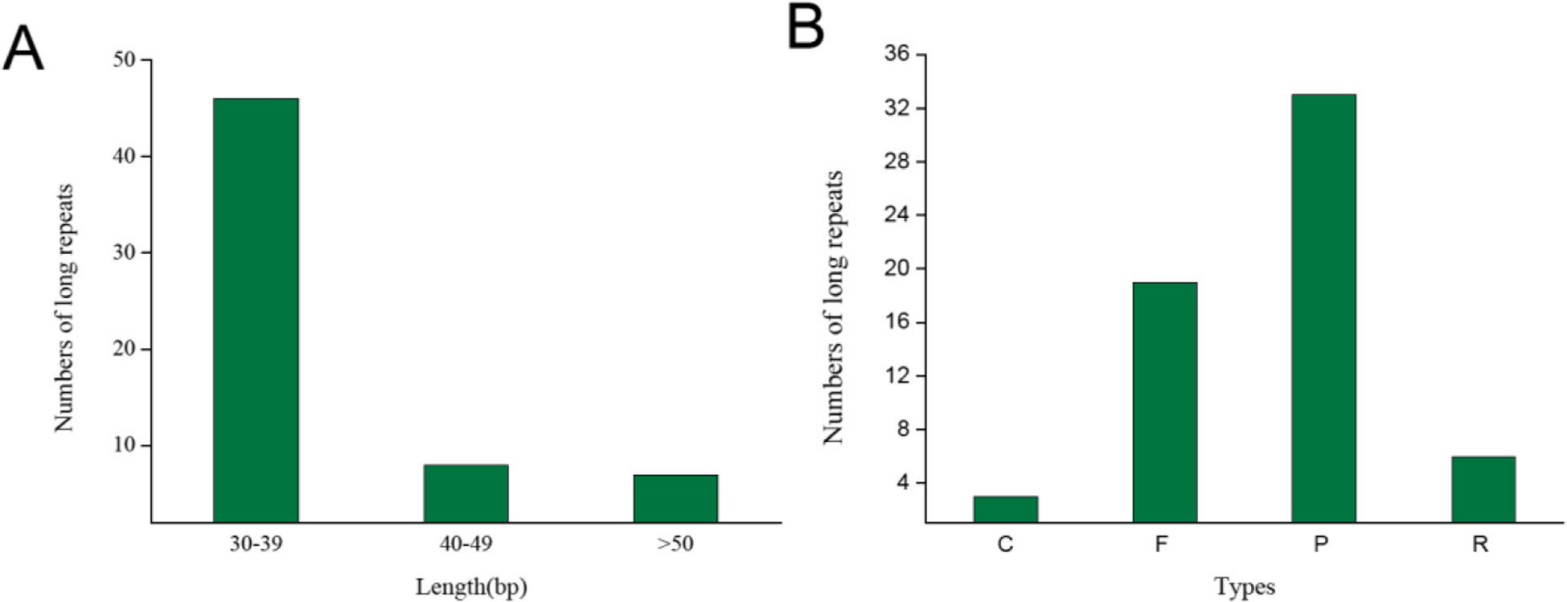
**a** Different lengths of long repeats, **b** Numbers of long repeats of different types. Note: P: palindromic repeats; F:forward repeats; R: reverse repeats; C: complement repeats

The analysis of long-repeat in the *P. lunatus* showed 33 palindromic repeats, 19 forward repeats, 6 reverse repeats and 3 complement repeats. Among them, 46 repeats were 30–39 bp in length, 8 repeats were 40–49 bp, 7 repeats were more than 50 bp, and the longest repeat was 287 bp in length and was located in the IR region (Fig. 2; Table S3). Most repeats were located in the intron sequences and intergenic spacer (IGS), and the minority were found in the *ycf2*, *rpl16*, *ndhA*, *ycf3*, *psbL*, *psaA*, *psaB*, *trnS-GGA*, *trnT-UGU*, *trnS-GCU*, *trnS-TGA*, *trnT-GGU*, *ndhF*, *trnS-GCU* and *trnK-UUU* genes.

**Fig. 3:**
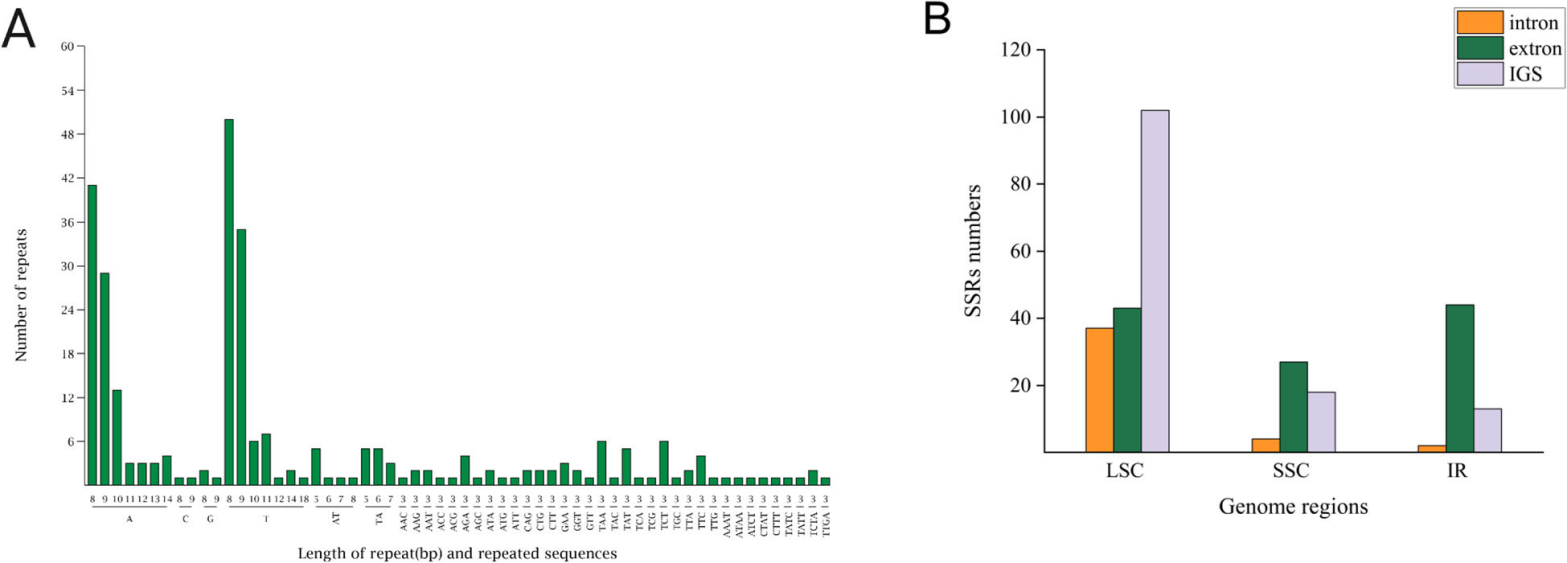
**a** Types and numbers of simple sequence repeats (SSRs) and **b** Simple sequence repeats (SSRs) distribution in different regions

Two hundred ninety SSRs were identified in *P. lunatus*, containing 203 mononucleotides, 21 dinucleotides, 56 trinucleotides, and 10 tetranucleotides (Fig. 3; Table S4). Among these SSRs, most distributed in LSC (63.45%) followed by SSC (22.76%) and IRs (13.79%), whereas 133 were located in intergenic spacers, 43 in introns and 114 in extrons, SSRs in genes including *ndhBA*\*DE*\*HF*, *ycf1–4*, *rpl1416*\*32133*, *ccsA*, *atpB*\*F*\*I*, *cemA*, *clpP*, *PetD*\*B*\*A*, *psaT*\*B*\*CA*, *rbcL*, *rp12*\*132*, *rpoA*\*B*\*C1*\*C2*, *rps2*\*14*\*15*\*18*\*19*, *rrn23*, *trnK-UUU* (intron)/*matK*, *trnK-UUU*, *trnV-UAC*, *trnG-UCC* and *trnI-GAU*.

### Gene order

**Fig. 4:**
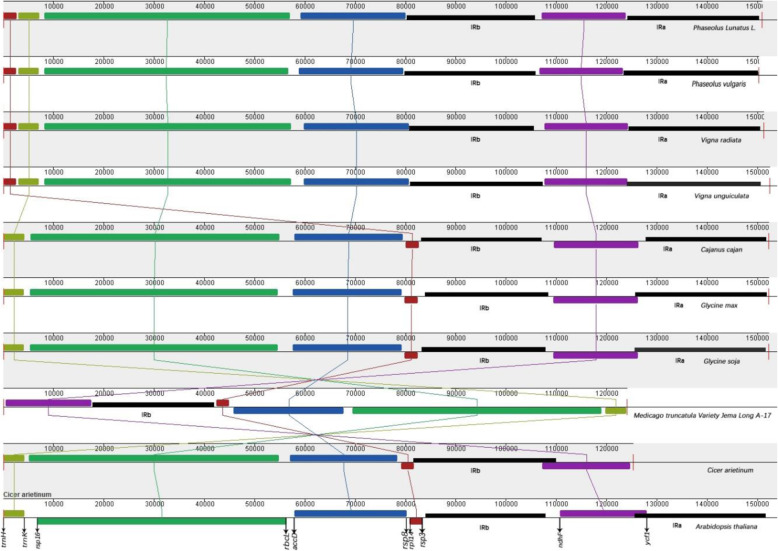
Gene order comparison of legume plastid genomes, using MAUVE software. The boxes above the line represent the gene sequence in the clockwise direction, and the boxes below the line represent gene sequences in the opposite orientation. The gene names at the bottom indicate the genes located at the boundaries of the boxes in the Cp genome of *Arabidopsis*

The Cp genome structures of eight-sequenced legumes were selected and compared with lima bean using Mauve software, with the of *A. thaliana* as a reference (Fig. 4). All the legume have almost the same gene order, and the Cp genomes of *C. arietinum* and *M. truncatula* have lost one copy of the IR. on comparison with Arabidopsis, all have a common 50-kb inversion, spanning from *rbcL* to *rps16* gene in the LSC region. The Cp genomes of *P. lunatus*, *P. vulgaris*, *V. radiata* and *V. unguicalata* have 70 kb inversion to subtribe Phaseolinae but are not found in other Cp genomes. *G soja*, *M. truncatula* and *C. arietinum* share the same gene order with *C. cajan*, *G. max* and *G soja* except for the loss of the IRb region.

### Comparison of complete chloroplast genomes among Leguminosae species

**Fig. 5:**
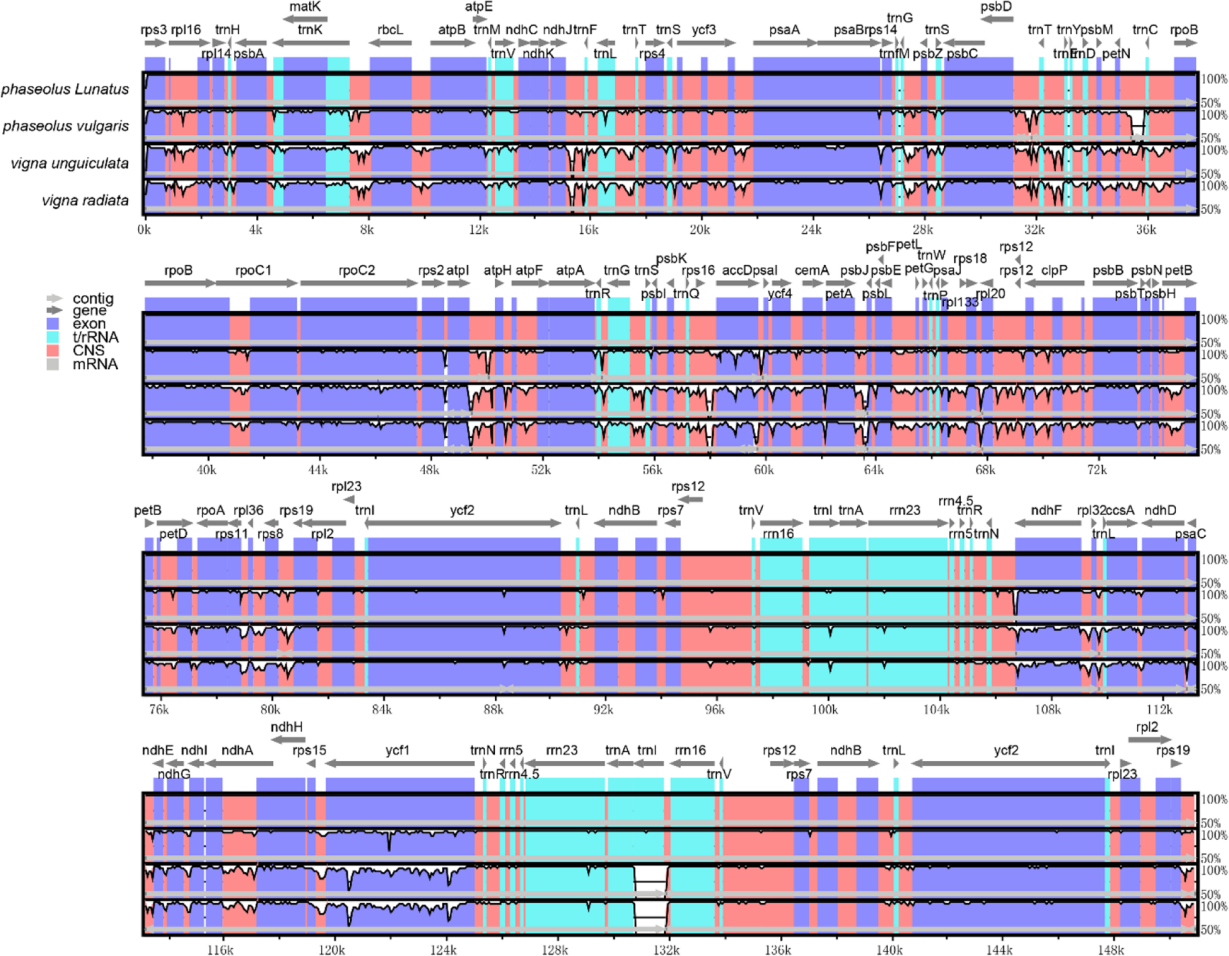
The comparison of four Phaseolinae species Cp genomes by using mVISTA. The grey arrows above the contrast indicate the direction of the gene translation. The y-axis represents the percent identity between 50 and 100%. Protein codes (exon), rRNAs, tRNAs and conserved noncoding sequences (CNSs) are shown in different colours

To verify the possibility of genome divergence, mVISTA was used to compare the Phaseolinae Cp genomes, using annotations of lima bean as a reference (Fig. 5). The result shows high sequence identity with Phaseolinae species. *rpl16*, *accD*, *petB*, *rsp16, clpP*, *ndhA, ndhF* and *ycf1* genes in coding regions was found with significant variation, *trnk*-*rbcL, rbcL*-*atpB*, *ndhJ*-*rps4*, *psbD*-*rpoB*, *atpI*-*atpA*, *atpA*-*accD*, *accD-psbJ, psbE*-*psbB*, *rsp11-rsp19*, *ndhF-ccsA* in the intergenic regions were identified with a high degree of divergence *.*

**Fig. 6:**
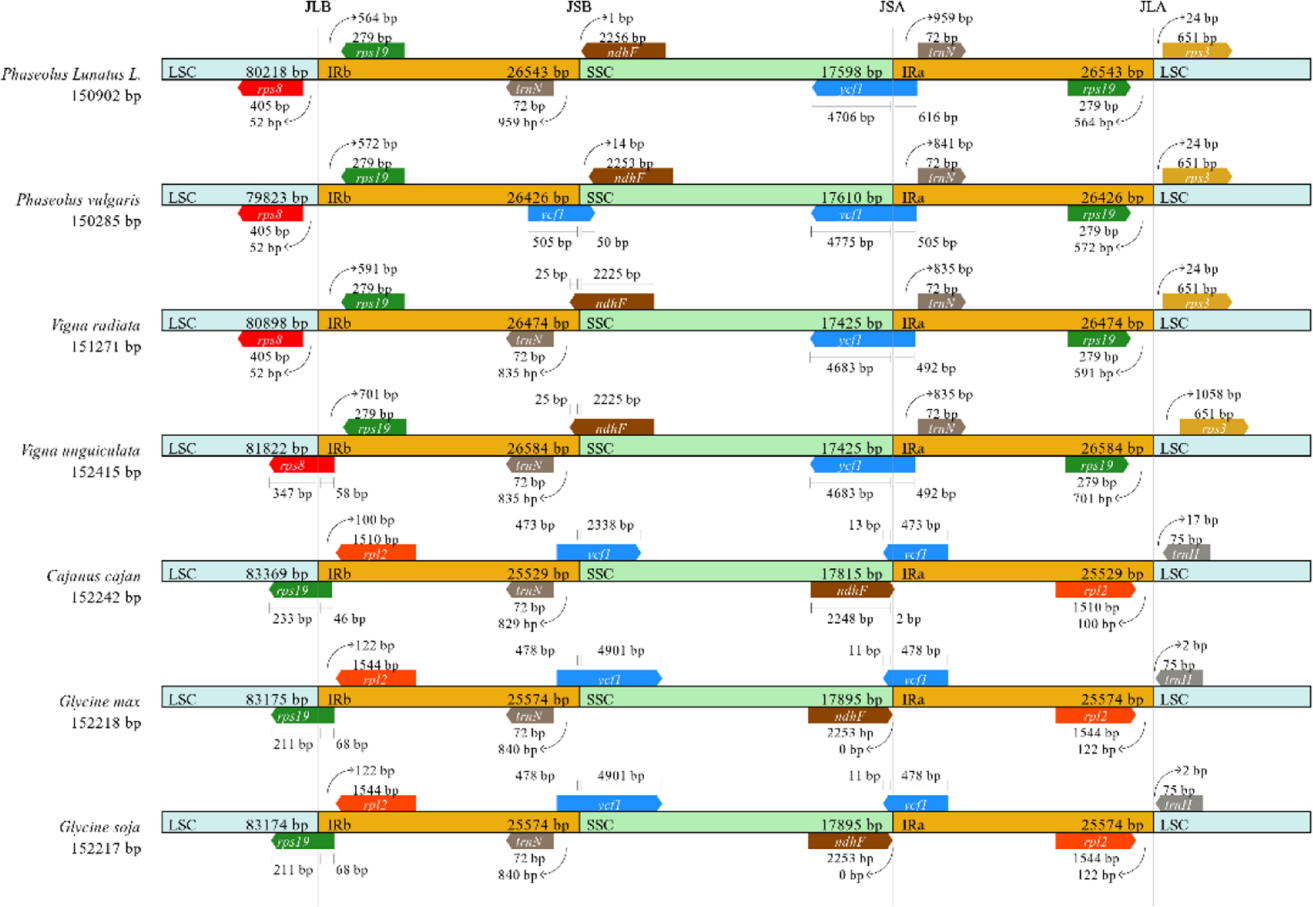
Comparison of the borders of the LSC and SSC regions and IRs among seven Leguminosae species. Genes are denoted by boxes, and the gaps between the genes and the boundaries are indicated by number of bases, unless the gene coincides with the boundary. Extensions of the genes are also indicated above the boxes

A comparison of the boundaries of the lima bean Cp genome was performed among the other six Leguminosae species: *P. vulgaris*, *V. radiata*, *V. unguiculata*, *C. cajan*, *G.max*, and *G. soja* (Fig. 6). At the LSC/IR junction of lima bean, the *rps19* and *trnN* genes are duplicated at the IR/SSC junction completely and included in the IR region. a partial *ycf1* gene is included at the IRa/SSC junction. Compared to other species in the genus, the range of each region showed substantial differences. The *rps19* gene in the *P. lunatus*, *P. vulgaris*, *V. radiate* Cp genomes was shifted by 564 bp from IR to LSC at the LSC/IR border and 701 bp from IR to LSC in the *V. unguiculata*. However, in *C. cajan*, *G. max* and *G. soja*, the *rps19* gene crossed the IRb/LSC region, with 46, 68 and 68 bp of *rps19* gene within IRb, respectively. On the other hand, the *ycf1* gene is located at the IRa/SSC border in all the compared legumes, but the junctions of IRa/SSC located in *ycf1* within the SSC and IRa regions vary in length (*P. lunatus*: 4706 and 616 bp; *P. vulgaris*: 4775 and 505 bp; *V. radiate*: 4683 and 492 bp; *V. unguiculata*: 4683 and 492 bp; *C. cajan*: 13 and 473 bp; *G.max*: 11 and 478 bp; *G. soja*: 11 and 478 bp), while the *ycf1* gene was only at the IRb/SSC border of *P. vulgaris*, *C. cajan*, *G. max*, and *G. soja* and the size varies among them.

### Adaptive evaluation analysis

**Fig. 7:**
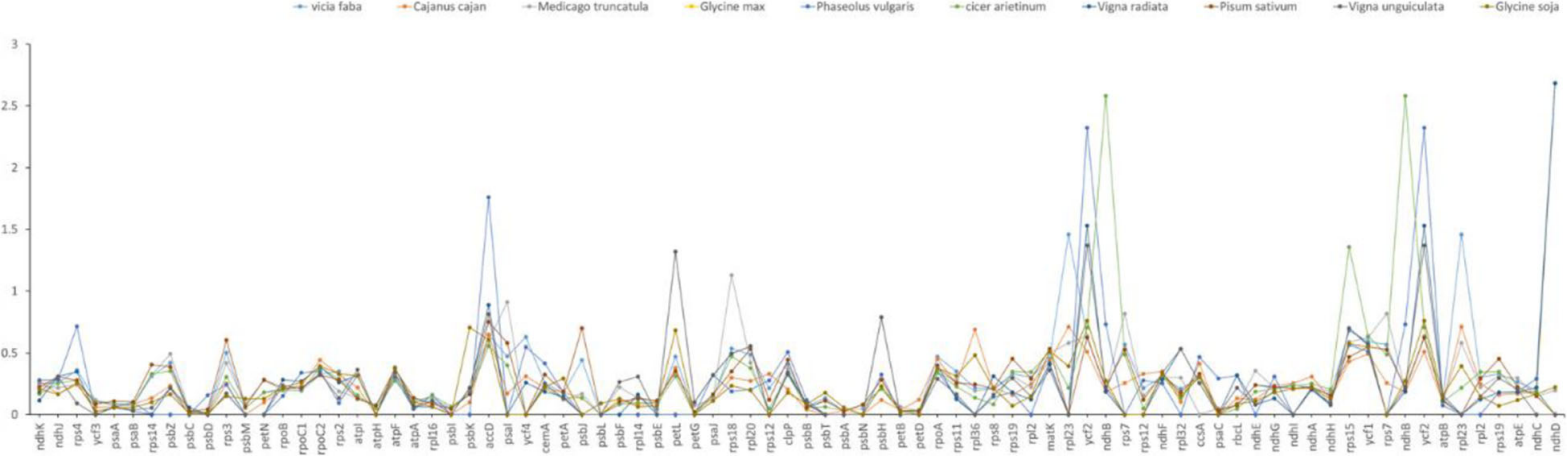
The Ka/Ks ration values of 82 protein coding genes from ten Leguminosae cp genomes

The Ka/Ks ratio were calculated by KaKs_Calculator among the Cp genome of eleven species of Leguminosae protein-coding genes. The results indicated that the Ka/Ks ratio is < 1 in mostly except for *rpl23* of *V. faba* vs *P. lunatusis*, *ndhD* of *C. cajan*, *rps18* of *M. truncatula* vs *P. lunatusis*, *ndhD* of *G. max* vs *P. lunatusis*, *accD/ ycf2/ ndhD* of *P. vulgaris* vs *P. lunatusis*, *ndhB*/ *rps15*/ *ndhB* of *C. arietinum* vs *P. lunatusis*, *petL*/ *ycf2*/ *ndhD* of *V radiata* vs *P. lunatusis*, *petL*/ *ycf2* of *V. unguiculata* vs *P. lunatusis* (Fig. 7). For each gene, the majority had a Ka/Ks ratio < 0.5 for the ten comparison groups. At the same time, 13 of them had a Ka/Ks ratio between 0 and 0.1. In contrast, the Ka/Ks ratio of the *ndhD* gene was greater than 1 in four of the ten comparison groups, four of them had no this gene and another two exhibited low Ka/Ks ratios. Moreover, *ycf2* also exhibited a Ka/Ks ratios > 1 in three of them and the ratio > 0.5 in the other species.

### Phylogenetic analysis

**Fig. 8:**
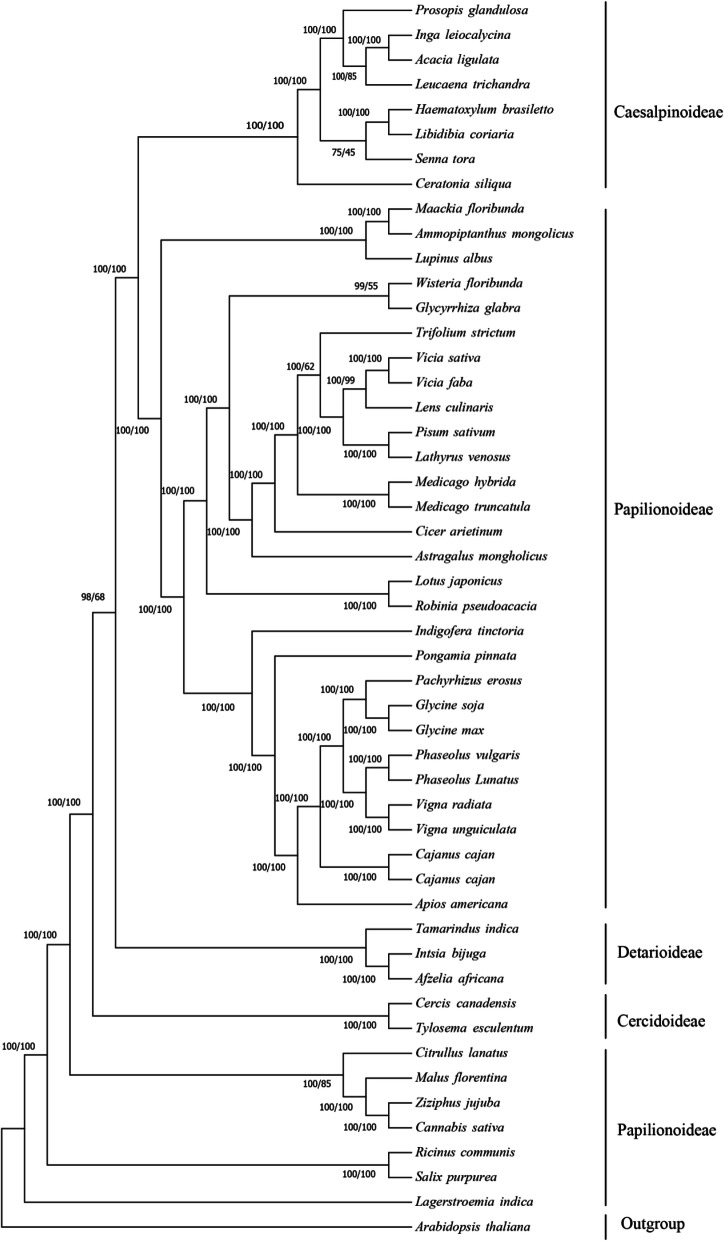
Maximum likelihood (ML) and Bayesian Inference (BI) phylogenetic tree of 48 species of Leguminosae constructed using the sequences of 44 proteins. *Arabidopsis thaliana* were used as the outgroups

To identified the phylogenetic position of lima bean in Leguminosae, we used the 44 protein sequences of 48 Leguminosae species to phylogenetic analyse (Fig. 8). Maximum likelihood (MI) and Bayesian inference (BI) were used to construct phylogenetic tree with *Arabidopsis thaliana* as outgroup. The phylogenetic results resolved most nodes with bootstrap support values of 100. These 48 species belong to Caesalpinoideae, Cercidoideae, Detarioideae and Papilionoideae. The phylogenetic tree showed that *P. lunatus* and *P. vulgaris* are sister spisecies with a 100% bootstrap value and *P. lunatus*is more closely related to *P. vulgaris*, *V. unguiculate* and *V. radiata.* The phylogenetic trees are very helpful for us to understand the phylogenetic relationship among more Leguminosae species.

## Discussion

In this study, the Cp genome of lima bean were sequenced and assembled, and this information was applied for their comparative analysis with other Leguminosae species. The size of genome, content of GC, the length of IR, LSC, and SSC regions and gene content exposed high similarity among the genomes, suggesting that leguminosae species shared low diversity [20, 21, 37, 38]. The GC content is closely related to species affinity [39]. High GC content is conducive to the stability of the genome and maintaining the complexity of the sequence. The four rRNAs genes have high GC content, which results in a high GC content in the IR region [40]. The codon usage bias is related to translational efficiency, which biased towards rich tRNA. At the same time, those codons that bind more tightly than other homologous tRNAs [41]. In this study, all genes were encoded by 25,873 codons, in these codons the most frequent amino acids are leucine (2719, 10.51%) and the least are cysteine (300, 1.16%). The most preferred synonymous codons end with A and U. High AT abundance is the main cause why synonym codons end with A/U, which may be the result of natural selection and mutation [42, 43].

Repeat sequences are significant important for genome rearrangements and variations, and repeat occurrence is more prevalent in IGSs than in genic sequences [44–46]. Furthermore, these repeats can be used to develop genetic markers for phylogeny and population studies [47]. We found 61 repeat sequences in *P. lunatus* Cp genome, and most of the repeats were distributed within the intergenic spacer regions, intron sequences, and *ycf2* genes, which is highly homologous to the sequence in *V.radiata* [20].

Currently, chloroplast genome markers have more advantages than nuclear DNA markers in terms of evolution and taxonomic research due to their maternal inheritance in most plants and much lower mutation rate [48, 49]. cpSSRs are often used to identify species and analyze genetic because they are relatively richness and have demonstrated high reproducibility and polymorphism. Two hundred ninety SSRs were found in the lima bean Cp genime. The number of SSR is similar to those in pigeonpea [37], but more than clusterbean [18]. Among 290 SSRs, most of them distributed in LSC (63.45%) and located in intergenic spacers (45.86%). The findings were similar to clusterbean [18] and pigeonpea [37].

Although the Cp genomes of angiosperms are well-conserved, inversion, rearrangement, novel DNA insertion and IR expansion contraction occur frequently [18, 21, 25]. Leguminosae is an excellent choice for studying the evolution of the Cp genome because legume plastid genomes have undergone multiple genomic rearrangements and the loss of genes or introns [50].. In our study, *P. lunatus* has a common 50 kb inversion in the LSC region, spanning from *rbcL* to *rps16*, which has been found in other legumes (Fig. 4) [18, 20, 21]. Due to the expansion and contraction of IRs, the Cp genomes of *P. vulgaris*, *V. radiata* and *V. unguicalata* have 70 kb inversion to subtribe Phaseolinae but are absent from other Cp genomes [51, 52]. *P. lunatus* as a member of the subtribe Phaseolinae shows the same inversion. All the results shown in the gene order suggest that considerable rearrangements and diversification were occurred in the legume Cp genomes and a valuable resource for phylogenetic analysis is provided.

Crop evolution and genetic improvement progress mainly depends on the genetic diversity available in germplasm resources [53]. *rpl16*, *accD*, *petB*, *rsp16, clpP*, *ndhA, ndhF* and *ycf1* genes in CDS was found significant variation and high sequence variations were found in intergenic regions as follows: *trnk*-*rbcL, rbcL*-*atpB*, *ndhJ*-*rps4*, *psbD*-*rpoB*, *atpI*-*atpA*, *atpA*-*accD*, *accD-psbJ, psbE*-*psbB*, *rsp11-rsp19*, *ndhF-ccsA* (Fig. 5)*.* These regions were considered useful markers for elucidating phylogenetic relationships among Leguminosae species. The *ycf1*, *accD* and *ndhF* genes were also served as genetic markers for *Quercus bawanglingensis* [54]. The Cp DNA regions: *trnL*-*trnF* and *atpB*-*rbcL* were used to evaluate 262 accessions of *P. lunatus* to identify whether the MA gene pool of *P. lunatus* has a single centre or multiple centres [55]. *trnL-trnF* in noncoding Cp DNA regions also has been used to study Phylogeny and domestication [56–58]. polymorphisms of the Cp DNA is very useful to study the evolutionary of Lima bean and to pinpoint domestication places in several studies. Hence, more and more genome resources need to be developed for plants [59].

Studies have shown that IR regions in plant chloroplast genomes are more conserved than single copy and non-coding regions, and can stable the rest genome [38]. The size change of the angiosperm plastid genome is caused by the contraction and expansion of the IR region at the boundary [51, 60]. The change of the IR/SC junction is a common phenomenon and plays an important role in evolution [54, 61, 62]. In the seven Leguminosae species, *P. lunatus*, *P. vulgars*, *V. radiate* and *V. unguiculata* showed similar characteristics, only some genes including *rps8*, *rps19*, *trnN*, *ndhF*, *ycf1* and *rps3* showed a little difference; *C. cajan*, *G.max*, and *G. soja* showed more differences than other four species (Fig. 6). The complete *trnH*-*rps19* cluster of *P. lunatus*, *P. vulgars*, *V. radiata* and *V. unguiculata* is present in IR regions, which is consistent with TYPE III [63].

Non-synonymous (Ka) and synonymous (Ks) substitutions and their ratios (Ka/Ks) have been used to assess the rate of gene divergence. The ratio of Ka/Ks < 1 represents purifying selection, while the ratio > 1 represents positive selection [64]. In most protein-coding genes, nucleotide substitutions of synonymous occur more frequently than non-synonymous [65]. In this study, the ratio is < 1 in most of the genes, indicating that they are under purifying selection in lima bean. However, the Ka/Ks ratio of *ndhD* gene is > 1 in four of the ten comparison groups, *ycf2* exhibited a ratios > 1 in three of them and the ratio > 0.5 in the other species. The *ndhD* and *ycf2* undergo positive selection in lima bean, which may help to adapt to their living environment.

Genetic analysis of lima bean was performed using cytogenetic [66] and molecular data [55]. With the development of sequencing technologies, an increasing number of Cp genomes have been used for phylogenetic analysis [35, 36]. The Cp genomes have been used for phylogenetic analyses in the genus *Quercus*, which provide strong support for the deep phylogenetic relationship between subfamily tribes [67].. In our study, the sequences of chloroplast genomes were used for phylogenetic analysis by ML and BI based on 48 Leguminosae species. *P. vulgaris* and *P. lunatus* are sister species, *P. lunatus* is more closely related to *P. vulgaris*, *V. unguiculata* and *V. radiata*. Consistent with the gene order results, they are all of subtribe *Phaseolinae*. The result is consistent with other phylogenies constructed by Cp genome containing representatives *Phaseolinae* genus [68–70].

## Conclusions

In this study, the complete Cp genome of *P.lunatus* was first sequenced on IlluminaNextera XT platforms. The size of genome, structure and organization of gene were shown to be conservative, which is similar to those reported Cp genomes of Leguminosae species. Sixty-one repeats and 290 SSRs were present in *P. lunatus*. These results are very useful for developing barcoding molecular markers. In comparison with other legume species, the Cp genome of lima bean shares a similar gene order and IR region borders with *P. vulgaris*, *V. unguiculata* and *V. radiata*. Phylogenetic analysis of 48 Leguminosae species shows that *P. lunatus* are more closely related to *P. vulgaris*, *V. unguiculata* and *V. radiata*. These results provide important information for the complete Cp genome of *P. lunatus*, which might be useful for further studies of evolution and phylogenetic.

## Methods

### Sequencing and assembly of lima bean Cp genome

Fresh leaves were collected from lima bean plants grown on Huiyuan Vegetable Gardening Farm at Chongming Island [6]. Genomic DNA was extracted by CTAB method [71]. Then the DNA quality was tested (> 50 ng·μL − 1). The DNA was sequenced by the HiSeq™ X10 platform (Illumina, USA) at Nanjing. Bowtie2 v2.2.4 [72] was used to exclude non-chloroplast genome reads with paired-end alignments and a maximum of 3 mismatches(−v = 3), as the raw sequence reads always include non-cpDNA. The Cp genome was assembled by SPAdesv3.10.1 [73] and with the options of “–trusted-contigs” via manual correction using comparison with the reference species *P. vulgaris* (NCBI ACCESSION NC_009259.1). The Cp genome of lima bean was submitted into GenBank (SRA: SRR13319750, BioProject: PRJNA688003 Accession number: MW423611).

### Genome annotation of the cp DNA sequences

The annotation of the Lima bean Cp genome was performed by blast v2.2.2 (parameter: -nproc 20, −bestn 5 [74]., and the final annotation result was correct manually. rRNAs and tRNAs were identified by hmmer v3.1b2 [75] and aragorn v1.2.38 [76], respectively. The entire genome was mapped by OGDRAW [77]. The synonymous codon usage, relative synonymous codon usage values (RSCU) and codon usage of the complete plastid genomes were analyzed using MEGA 6.0 PREP suit [78] with cut off values of 8.0 was used to predict the RNA editing sites in the plastome.

### Characterization of repeat sequences and SSRs

Interspersed repeated sequences were detected by Vmatch v2.3.0 [79]. Simple sequence repeats (SSRs) were identified by MISA v1.0 [80].

### Comparative analysis of Cp genomes

MUMmer was used to pair sequence alignment of the chloroplast genome [81]. The chloroplast genome of *P. lunatus* (SRA: SRR13319750, BioProject: PRJNA688003) was compared with *P. vulgaris* (NC_009259), *V. radiate* (NC_013843) and *V. unguiculata* (NC_018051) in the Leguminosae tribe by mVISTA with the shuffle-LAGAN mode [82, 83]. The annotation of *P. lunatus* was set as a reference.

The gene order comparison was performed by MAUVE [84] between lima bean (SRA: SRR13319750, BioProject: PRJNA688003), *Arabidopsis thaliana* (NC_000932), *C. cajan* (KU729879), *G. max* (NC_007942), *P. vulgaris* (NC_009259), *C. arietinum* (NC_011163), *V. radiate* (NC_013843), *G. soja* (NC_022868), *V. unguiculata* (NC_018051) and *M. truncatula* (NC_003119).

### Adaptive evaluation analysis

In order to analyze non-synonymous (Ka) and synonymous (Ks) substitution rates and Ka/Ks ratio, *P. lunatus* was compared with the ten other species in Leguminosae tribe: *G. max*, *C. cajan*, *C. arietinum*, *V. radiate*, *P. vulgaris*, *G. soja*, *V. unguiculata, P. sativum*, *V. faba* and *M. truncatula*. The ten sequences was separately aligned by MAFFT v7.427 [85], then the Ka and Ks substitution rates and Ka/Ks value was counted using the KaKs_calculator 2.0 [86] with the default model averaging (MA) method.

### Phylogenetic analysis

The phylogenetic analysis was conducted for lima bean, another 47 Leguminosae species, and one outgroup *Arabidopsis thaliana*, all of which were down loaded from the NCBI except those of *P. lunatus*. The complete Cp genomes were aligned using MAFFT v7.427 [85]. RAxML v.8.2.10 [87] and MrBayes version 3.2.6 [88] was used to reconstruct the phylogenetic relationship with the maximum likelihood (ML) and Bayesian Inference (BI) methods.

### Availability of data and materials

The Cp genome of *P. lunatus* were uploaded to the NCBI database (https://www.ncbi.nlm.nih.gov/) with GenBank accession numbers (SRA: SRR13319750, BioProject: PRJNA688003). Other data can be obtained by contacting the corresponding author.

## Supplementary Information

**Additional file 1: Table S1.** The number of genes in the *P. lunatus* Cp genome.

**Additional file 2: Table S2.** The relative synonymous codon usage of the *P. lunatus* chloroplast genome.

**Additional file 3: Table S3.** Repeated sequences of the *P. lunatus* chloroplast genome.

**Additional file 4: Table S4.** Simple sequence repeats (SSRs) in the *P. lunatus* chloroplast genome.

## Data Availability

The datasets supporting the results of this publication are included within the article and Additional files 1, 2, 3, 4.

## Abbreviations

CNS: Conserved non coding sequence
Cp: Chloroplast
DNA: Deoxyribonucleic acid
IGS: Intergenic spacer
IR: Inverted repeat
LSC: Large single copy region
RSCU: Relative synonymous codon usage
SSC: Small single copy region
SSR: Simple sequence repeats
Ka: Non-synonymous; Ks: synonymous

## References

[1] Jean-pierre Baudoin OR, Degreef J, Maquet A, Guarino L. Ecogeography, demography, diversity and conservation of *Phaseolus lunatus* L. in the central valley of Costa Rica. Systematic & Ecogeographic Studies on Crop Genepools. 2004. p. 1-94.

[2] Almeida C, Pedrosa-Harand A (2013). High macro-collinearity between lima bean (Phaseolus lunatus L.) and the common bean (P. vulgaris L.) as revealed by comparative cytogenetic mapping. Theor Appl Genet.

[3] Chacon-Sanchez MI, Martinez-Castillo J (2017). Testing Domestication Scenarios of Lima Bean (*Phaseolus lunatus* L.) in Mesoamerica: Insights from Genome-Wide Genetic Markers. Front Plant Sci.

[4] Bi IZ, Maquet A, Baudoin JP (2003). Population genetic structure of wild *Phaseolus lunatus* (Fabaceae), with special reference to population sizes. Am J Bot.

[5] Zoro BI, Maquet A, Degreef J, Wathelet BJP. BaudoinSample size for collecting seeds in germplasm conservation: the case of the Lima bean (*Phaseolus lunatus L.*). Theor Appl Genet. 1998;97(1-2):187-94.

[6] Rong-Fei MA, Fan-Lei M, Li-Jun GU (2013). Cluster analysis and evaluationon germplasm resources of Chongming lima bean. Acta Agriculturae Shanghai.

[7] Rono PC, Dong X, Yang JX, Mutie FM, Oulo MA, Malombe I, Kirika PM, Hu GW, Wang QF (2020). Initial complete chloroplast genomes of Alchemilla (Rosaceae): comparative analysis and phylogenetic relationships. Front Genet.

[8] Jansen RK, Wojciechowski MF, Sanniyasi E, Lee SB, Daniell H (2008). Complete plastid genome sequence of the chickpea (Cicer arietinum) and the phylogenetic distribution of rps12 and clpP intron losses among legumes (Leguminosae). Mol Phylogenet Evol.

[9] Yue F, Cui L, Depamphilis CW, Moret BME, Tang J (2008). Gene rearrangement analysis and ancestral order inference from chloroplast genomes with inverted repeat. BMC Genomics.

[10] Aldrich J, Cherney B, Merlin E, Williams C, Mets L (1985). Recombination within the inverted repeat sequences of the Chlamydomonas reinhardii chloroplast genome produces two orientation isomers. Curr Genet.

[11] Aldrich J, Cherney BW, Williams C, Merlin E (1988). Sequence-analysis of the junction of the large single copy region and the large inverted repeat in the petunia chloroplast genome. Curr Genet.

[12] Chang CC, Lin HC, Lin IP, Chow TY, Chen HH, Chen WH, Cheng CH, Lin CY, Liu SM, Chang CC (2006). The chloroplast genome of Phalaenopsis aphrodite (Orchidaceae): comparative analysis of evolutionary rate with that of grasses and its phylogenetic implications. Mol Biol Evol.

[13] Jansen RK (2011). Extreme reconfiguration of plastid genomes in the angiosperm family Geraniaceae: rearrangements, repeats, and codon usage. Mol Biol Evol.

[14] Huang S, Ge X, Cano A, Salazar BGM, Deng Y (2020). Comparative analysis of chloroplast genomes for five Dicliptera species (Acanthaceae): molecular structure, phylogenetic relationships, and adaptive evolution. PeerJ.

[15] Azani N, Babineau M, Bailey CD, Banks H, Barbosa AR, Pinto RB, Boatwright JS, Borges LM, Brown GK, Bruneau A et al. A new subfamily classification of the Leguminosae based on a taxonomically comprehensive phylogeny. Taxon. 2017;66(1):44-77.

[16] Gao CW, Gao LZ (2017). The complete chloroplast genome sequence of semi-wild soybean, Glycine gracilis (Fabales: Fabaceae). Conserv Genet Resour.

[17] Tomohiko K, Takakazu K, Shusei S, Yasukazu N, Satoshi T. Complete Structure of the Chloroplast Genome of a Legume, Lotus japonicus. DNA Research 2000;7:323–30. 10.1093/dnares/7.6.323 11214967

[18] Kaila T, Chaduvla PK, Rawal HC, Saxena S, Tyagi A, Mithra SVA, Solanke AU, Kalia P, Sharma TR, Singh NK (2017). Chloroplast Genome Sequence of Clusterbean (*Cyamopsis tetragonoloba* L.): Genome Structure and Comparative Analysis. Genes.

[19] Saski C, Lee SB, Daniell H, Wood TC, Tomkins J, Kim HG, Jansen RK (2005). Complete chloroplast genome sequence of Glycine max and comparative analyses with other legume genomes. Plant Mol Biol.

[20] Tangphatsornruang S, Sangsrakru D, Chanprasert J, Uthaipaisanwong P, Yoocha T, Jomchai N, Tragoonrung S (2009). The Chloroplast Genome Sequence of Mungbean (*Vigna radiata*) Determined by High-throughput Pyrosequencing: Structural Organization and Phylogenetic Relationships. DNA Research.

[21] Guo XW, Castillo-Ramirez S, Gonzalez V, Bustos P, Fernandez-Vazquez JL, Santamaria RI, Arellano J, Cevallos MA, Davila G (2007). Rapid evolutionary change of common bean (Phaseolus vulgaris L) plastome, and the genomic diversification of legume chloroplasts. BMC Genomics.

[22] Lavin M, Herendeen PS, Wojciechowski MF, Lavin M, Herendeen PS, Wojciechowski MF (2005). Evolutionary rates analysis of Leguminosae implicates a rapid diversification of lineages during the tertiary. Syst biol 54: 530-549. Syst Biol.

[23] Palmer JD, Thompson WF (1982). Chloroplast DNA rearrangements are more frequent when a large inverted repeat sequence is lost. Cell.

[24] Lavin M, Doyle JJ, Palmer JD (1990). EVOLUTIONARY SIGNIFICANCE OF THE LOSS OF THE CHLOROPLAST-DNA INVERTED REPEAT IN THE LEGUMINOSAE SUBFAMILY PAPILIONOIDEAE. Evolution.

[25] Cai ZQ, Guisinger M, Kim HG, Ruck E, Blazier JC, McMurtry V, Kuehl JV, Boore J, Jansen RK (2008). Extensive reorganization of the plastid genome of Trifolium subterraneum (Fabaceae) is associated with numerous repeated sequences and novel DNA insertions. J Mol Evol.

[26] Doyle JJ, Doyle JL, Palmer JD (1995). Multiple independent losses of two genes and one intron from legume chloroplast genomes. Syst Bot.

[27] Gantt JS, Baldauf SL, Calie PJ, Weeden NF, Palmer JD (1991). Transfer of rpl22 to the nucleus greatly preceded its loss from the chloroplast and involved the gain of an intron. EMBO J.

[28] Magee AM, Aspinall S, Rice DW, Cusack BP, Semon M, Perry AS, Stefanovic S, Milbourne D, Barth S, Palmer JD (2010). Localized hypermutation and associated gene losses in legume chloroplast genomes. Genome Res.

[29] Moore MJ, Soltis PS, Bell CD, Burleigh JG, Soltis DE (2010). Phylogenetic analysis of 83 plastid genes further resolves the early diversification of eudicots. Proc Natl Acad Sci U S A.

[30] Dan Z, Kui L, Ju G, Yuan L, Li-Zhi G (2016). The complete plastid genome sequence of the wild Rice Zizania latifolia and comparative chloroplast genomics of the Rice tribe Oryzeae, Poaceae. Front Ecol Evol.

[31] Osuna-Mascaró C, Rafael RDC, Perfectti F (2018). Comparative assessment shows the reliability of chloroplast genome assembly using RNA-seq. Sci Rep.

[32] Käss E, Wink M (1997). Phylogenetic relationships in the Papilionoideae (family Leguminosae) based on nucleotide sequences of cpDNA (rbcL) and ncDNA (ITS 1 and 2). Mol Phylogenet Evol.

[33] Brouat C, Gielly L, McKey D (2001). Phylogenetic relationships in the genus Leonardoxa (Leguminosae: Caesalpinioideae) inferred from chloroplast trnL intron and trnL-trnF intergenic spacer sequences. Am J Bot.

[34] Manzanilla V, Bruneau A (2012). Phylogeny reconstruction in the Caesalpinieae grade (Leguminosae) based on duplicated copies of the sucrose synthase gene and plastid markers. Mol Phylogenet Evol.

[35] Alzahrani DA, Yaradua SS, Albokhari EJ, Abba A (2020). Complete chloroplast genome sequence of Barleria prionitis, comparative chloroplast genomics and phylogenetic relationships among Acanthoideae. BMC Genomics.

[36] Lemieux C, Otis C, Turmel M (2016). Comparative chloroplast genome analyses of Streptophyte Green algae uncover major structural alterations in the Klebsormidiophyceae, Coleochaetophyceae and Zygnematophyceae. Front Plant Sci.

[37] Kaila T, Chaduvla PK, Saxena S, Bahadur K, Gahukar SJ, Chaudhury A, Sharma TR, Singh NK, Gaikwad K. Chloroplast Genome Sequence of Pigeonpea (*Cajanus cajan* (L.) Millspaugh) and *Cajanus scarabaeoides* (L.) Thouars: Genome Organization and Comparison with Other Legumes. Front Plant Sci. 2016;7:1847. 10.3389/fpls.2016.01847 PMC5145887 28018385

[38] Kaila T, Chaduvla PK, Saxena S, Bahadur K, Gahukar SJ, Chaudhury A, Sharma TR, Singh NK, Gaikwad K. Chloroplast Genome Sequence of Pigeonpea (*Cajanus cajan* (L.) Millspaugh) and *Cajanus scarabaeoides* (L.) Thouars: Genome Organization and Comparison with Other Legumes. Front Plant Sci. 2016;7:1847. 10.3389/fpls.2016.01847 PMC5145887 28018385

[39] Budhi DA, Yohei T, Sri S, Arifin ZMS, Toyoko A, Yoko S, Petr H (2017). The origin and evolution of fibromelanosis in domesticated chickens: genomic comparison of Indonesian Cemani and Chinese Silkie breeds. PLoS One.

[40] Kaila T, Chaduvla PK, Saxena S, Bahadur K, Gahukar SJ, Chaudhury A, Sharma TR, Singh NK, Gaikwad K (2016). Chloroplast Genome Sequence of Pigeonpea (*Cajanus cajan* (L.) Millspaugh) and *Cajanus scarabaeoides* (L.) Thouars: Genome Organization and Comparison with Other Legumes. Front Plant Sci.

[41] Salim HMW, Cavalcanti ARO (2008). Factors influencing codon usage bias in genomes. J Braz Chem Soc.

[42] Necşulea A, Lobry JR (2007). A new method for assessing the effect of replication on DNA base composition asymmetry. Mol Biol Evol.

[43] Shimada H, Sugiura M (1991). Fine structural features of the chloroplast genome: comparison of the sequenced chloroplast genomes. Nucleic Acids Res.

[44] Yan C, Du J, Gao L, Li Y, Hou X (2019). The complete chloroplast genome sequence of watercress (*Nasturtium officinale* R. Br.): Genome organization, adaptive evolution and phylogenetic relationships in Cardamineae. Gene.

[45] Su-Young H, Kyeong-Sik C, Ki-Oug Y, Hyun-Oh L, Kwang-Soo C, Jong-Taek S, Su-Jeong K, Jeong-Hwan N, Hwang-Bae S, Yul-Ho K (2017). Complete Chloroplast Genome Sequences and Comparative Analysis of Chenopodium quinoa and C album. Front Plant Sci.

[46] Huang YY, Matzke AJM, Matzke M (2013). Complete sequence and comparative analysis of the chloroplast genome of coconut palm (Cocos nucifera). PLoS One.

[47] Sajjad A, Muhammad W, Khan AL, Khan MA, Kang SM, Imran QM, Raheem S, Saqib B, Yun BW, In-Jung L (2017). The complete chloroplast genome of wild Rice (Oryza minuta) and its comparison to related species.

[48] Holwerda BC, Jana S, Crosby WL (1986). Chloroplast and mitochondrial DNA variation in HORDEUM VULGARE and HORDEUM SPONTANEUM. Genetics.

[49] Vanichanon A, Blake N, Sherman J, Talbert L (2003). Multiple origins of allopolyploid Aegilops triuncialis. Theor Appl Genet.

[50] Jansen RK, Cai Z, Raubeson LA, Daniell H, Depamphilis CW, Leebens-Mack J, Muller KF, Guisinger-Bellian M, Haberle RC, Hansen AK (2007). Analysis of 81 genes from 64 plastid genomes resolves relationships in angiosperms and identifies genome-scale evolutionary patterns. Proc Natl Acad Sci U S A.

[51] Tanvi K, Chaduvla PK, Swati S, Kaushlendra B, Gahukar SJ, Ashok C, Sharma TR, Singh NK, Kishor G (2016). Chloroplast Genome Sequence of Pigeonpea (*Cajanus cajan* (L.) Millspaugh) and *Cajanus scarabaeoides* (L.) Thouars: Genome Organization and Comparison with Other Legumes. Front Plant Sci.

[52] Bruneau A, Palmer DJD (1990). A chloroplast DNA inversion as a subtribal character in the Phaseoleae (Leguminosae). Syst Bot.

[53] Baudoin JP (1988). Genetic resources, domestication and evolution of lima bean, *Phaseolus lunatus*. J Emerg Med.

[54] Liu X, Chang E-M, Liu J-F, Huang Y-N, Wang Y, Yao N, Jiang Z-P (2019). Complete Chloroplast Genome Sequence and Phylogenetic Analysis of Quercus bawanglingensis Huang, Li et Xing, a Vulnerable Oak Tree in China. Forests.

[55] Andueza-Noh RH, Serrano-Serrano ML, Sánchez MC, Del Pino IS, Camacho-Pérez L, Coello-Coello J, Cortes JM, Debouck DG, Martínez-Castillo J (2013). Multiple domestications of the Mesoamerican gene pool of lima bean (Phaseolus lunatus L.): evidence from chloroplast DNA sequences. Genetic Resour Crop Evol.

[56] Taberlet P, Gielly L, Pautou G, Bouvet J (1991). Universal primers for amplification of three non-coding regions of chloroplast DNA. Plant Mol Biol.

[57] Shaw J, Lickey EB, Beck JT, Farmer SB, Liu WS, Miller J, Siripun KC, Winder CT, Schilling EE, Small RL (2005). The tortoise and the hare II: relative utility of 21 noncoding chloroplast DNA sequences for phylogenetic analysis. Am J Bot.

[58] Shaw J, Lickey EB, Schilling EE, Small RL (2007). Comparison of whole chloroplast genome sequences to choose noncoding regions for phylogenetic studies in angiosperms: the tortoise and the hare III. Am J Bot.

[59] Sánchez MIC (2017). Organelle genomes in Phaseolus beans and their use in evolutionary studies.

[60] Xiaohong Y (2015). The first complete chloroplast genome sequences in Actinidiaceae: genome structure and comparative analysis. PLoS One.

[61] Davis JI, Soreng RJ (2010). Migration of endpoints of two genes relative to boundaries between regions of the plastid genome in the grass family (POACEAE). Am J Bot.

[62] Huo YM, Gao LM, Liu BJ, Yang YY, Wu X (2019). Complete chloroplast genome sequences of four Allium species: comparative and phylogenetic analyses. Sci Rep.

[63] Wang RJ, Cheng CL, Chang CC, Wu CL, Su TM, Chaw SM (2008). Dynamics and evolution of the inverted repeat-large single copy junctions in the chloroplast genomes of monocots. BMC Evol Biol.

[64] Yang Z, Nielsen R (2000). Estimating synonymous and nonsynonymous substitution rates under realistic evolutionary models. Mol Biol Evol.

[65] Makalowski W, Boguski MS (1998). Evolutionary parameters of the transcribed mammalian genome: an analysis of 2,820 orthologous rodent and human sequences. Proc Natl Acad Sci U S A.

[66] Bonifácio EM, Fonsêca A, Almeida C, Santos KGBD, Pedrosa-Harand A (2012). Comparative cytogenetic mapping between the lima bean (Phaseolus lunatus L.) and the common bean (P. vulgaris L.). Theor Appl Genet.

[67] Xuan L, Yongfu L, Mingyue Z, Mingzhi L, Yanming F (2018). Complete Chloroplast Genome Sequence and Phylogenetic Analysis of *Quercus acutissima*. Int J Mol Sci.

[68] Zha X, Wang X, Li J, Gao F, Zhou Y (2020). Complete chloroplast genome of Sophora alopecuroides (Papilionoideae): molecular structures, comparative genome analysis and phylogenetic analysis. J Genet.

[69] Antunes AM, Soares TN, Targueta CP, Novaes E, Telles MP (2020). The chloroplast genome sequence of Dipteryx alata Vog. (Fabaceae: Papilionoideae): genomic features and comparative analysis with other legume genomes. Brazilian J Bot.

[70] Deng CY, Xin GL, Zhang JQ, Zhao DM (2019). Characterization of the complete chloroplast genome of Dalbergia hainanensis (Leguminosae), a vulnerably endangered legume endemic to China. Conserv Genet Resour.

[71] Doyle J. A rapid DNA isolation procedure for small quantities of fresh leaf tissue. Phytochem Bull. 1987;19:11–5.

[72] Langmead B, Salzberg SL. Fast gapped-read alignment with Bowtie 2. Nat Methods. 2012;9(4):357-9. 10.1038/nmeth.1923 PMC3322381 22388286

[73] Bankevich A, Nurk S, Antipov D, Gurevich AA, Dvorkin M, Kulikov AS, Lesin VM, Nikolenko SI, Pham S, Prjibelski AD (2012). SPAdes: a new genome assembly algorithm and its applications to single-cell sequencing. J Comput Biol.

[74] Christiam Camacho GC, Avagyan V, Ma N, Papadopoulos J, Bealer K, Madden TL (2009). BLAST+: architecture and applications. BMC Bioinformatics.

[75] Eddy SR, Eddy S (2015). HMMER: biosequence analysis using profile hidden Markov models.

[76] Nelson MJ, Dang Y, Filek E, Zhang Z, Yu VWC, Ishida KI, Green BR (2007). Identification and transcription of transfer RNA genes in dinoflagellate plastid minicircles. Gene.

[77] Lohse M, Drechsel O, Bock R (2007). OrganellarGenomeDRAW (OGDRAW): a tool for the easy generation of high-quality custom graphical maps of plastid and mitochondrial genomes. Curr Genet.

[78] Kurtz S, Choudhuri JV, Ohlebusch E, Schleiermacher C, Stoye J, Giegerich R (2001). REPuter: the manifold applications of repeat analysis on a genomic scale. Nucleic Acids Res.

[79] Kurtz S (2010). The Vmatch large scale sequence analysis software-a manual. Center Bioinformatics.

[80] Thiel T, Michalek W, Varshney RK, Graner A (2003). Exploiting EST databases for the development and characterization of gene-derived SSR-markers in barley ( Hordeum vulgare L.). Theor Appl Genet.

[81] Kurtz S, Phillippy AM, Delcher AL, Smoot ME, Shumway M, Antonescu C, Salzberg SL (2004). Versatile and open software for comparing large genomes. Genome Biol.

[82] Mayor C, Brudno M, Schwartz JR, Poliakov A, Rubin EM, Frazer KA, Pachter L, Dubchak I (2000). VISTA : visualizing global DNA sequence alignments of arbitrary length. Bioinformatics.

[83] Frazer KA, Pachter L, Poliakov A, Rubin EM, Dubchak I (2004). VISTA: computational tools for comparative genomics. Nucleic Acids Res.

[84] Darling AE, Mau B, Blattner FR, Perna NT (2004). Mauve: multiple alignment of conserved genomic sequence with rearrangements. Genome Res.

[85] Katoh K, Standley DM (2013). MAFFT multiple sequence alignment software version 7: improvements in performance and usability. Mol Biol Evol.

[86] Wang D, Zhang Y, Zhang Z, Zhu J, Yu J (2010). KaKs_Calculator 2.0: a toolkit incorporating gamma-series methods and sliding window strategies. Genomics Proteomics Bioinformatics.

[87] Stamatakis A (2014). RAxML version 8: a tool for phylogenetic analysis and post-analysis of large phylogenies. Bioinformatics.

[88] Ronquist F, Teslenko M, van der Mark P, Ayres DL, Darling A, Höhna S, Larget B, Liu L, Suchard MA, Huelsenbeck JP (2012). MrBayes 3.2: efficient Bayesian phylogenetic inference and model choice across a large model space. Syst Biol.

