# Supplementary material for: Chloroplast genome sequence of Chongming lima bean (Phaseolus lunatus L.) and comparative analyses with other legume chloroplast genomes
Source: BMC Genomics. 2021 Mar 18;22:194. doi: 10.1186/s12864-021-07467-8 (PMC7977240; doi:10.1186/s12864-021-07467-8)
Supplement: Supplementary file 1 — Additional file 1: Table S1. The number of genes in the P. lunatus Cp genome. [file 12864_2021_7467_MOESM1_ESM.docx]

Table S1. The number of genes in the *P. lunatus* Cp genome.

| Region | Number of CDS | Number of tRNAs | Number of rRNAs | Total |
| --- | --- | --- | --- | --- |
| LSC | 56 | 23 | 0 | 79 |
| SSC | 12 | 1 | 0 | 13 |
| IRA | 7 | 7 | 4 | 18 |
| IRB | 6 | 7 | 4 | 17 |
