# Supplementary material for: Chloroplast genome sequence of Chongming lima bean (Phaseolus lunatus L.) and comparative analyses with other legume chloroplast genomes
Source: BMC Genomics. 2021 Mar 18;22:194. doi: 10.1186/s12864-021-07467-8 (PMC7977240; doi:10.1186/s12864-021-07467-8)
Supplement: Supplementary file 2 — Additional file 2: Table S2. The relative synonymous codon usage of the P. lunatus chloroplast genome. [file 12864_2021_7467_MOESM2_ESM.docx]

Table S2. The relative synonymous codon usage of the *P. Lunatus* chloroplast genome.

| Amino acid | Codon | count | RSCU | tRNA | Amino acid | Codon | count | RSCU | tRNA |
| --- | --- | --- | --- | --- | --- | --- | --- | --- | --- |
| Ter | UAA | 47 | 1.7196 |  | Met | GUG | 2 | 0.0102 |  |
| Ter | UAG | 22 | 0.8049 |  | Met | UUG | 1 | 0.0051 | trnL-CAA |
| Ter | UGA | 13 | 0.4755 |  | Asn | AAC | 289 | 0.4412 | trnN-GUU |
| Ala | GCA | 373 | 1.1548 | trnA-UGC | Asn | AAU | 1021 | 1.5588 |  |
| Ala | GCC | 194 | 0.6008 |  | Pro | CCA | 317 | 1.2408 | trnP-UGG |
| Ala | GCG | 113 | 0.35 |  | Pro | CCC | 182 | 0.7124 |  |
| Ala | GCU | 612 | 1.8948 |  | Pro | CCG | 116 | 0.454 |  |
| Cys | UGC | 80 | 0.5334 | trnC-GCA | Pro | CCU | 407 | 1.5928 |  |
| Cys | UGU | 220 | 1.4666 |  | Gln | CAA | 738 | 1.5752 | trnQ-UUG |
| Asp | GAC | 178 | 0.3574 | trnD-GUC | Gln | CAG | 199 | 0.4248 |  |
| Asp | GAU | 818 | 1.6426 |  | Arg | AGA | 459 | 1.893 | trnR-UCU |
| Glu | GAA | 1012 | 1.5486 | trnE-UUC | Arg | AGG | 146 | 0.6018 |  |
| Glu | GAG | 295 | 0.4514 |  | Arg | CGA | 337 | 1.3896 |  |
| Phe | UUC | 505 | 0.6246 | trnF-GAA | Arg | CGC | 77 | 0.3174 |  |
| Phe | UUU | 1112 | 1.3754 |  | Arg | CGG | 96 | 0.396 |  |
| Gly | GGA | 701 | 1.6496 | trnG-UCC | Arg | CGU | 340 | 1.4022 | trnR-ACG |
| Gly | GGC | 158 | 0.3716 |  | Ser | AGC | 100 | 0.3018 | trnS-GCU |
| Gly | GGG | 249 | 0.586 |  | Ser | AGU | 402 | 1.2126 |  |
| Gly | GGU | 592 | 1.3928 |  | Ser | UCA | 430 | 1.2972 |  |
| His | CAC | 129 | 0.4196 | trnH-GUG | Ser | UCC | 294 | 0.8868 | trnS-GGA |
| His | CAU | 486 | 1.5804 |  | Ser | UCG | 181 | 0.546 |  |
| Ile | AUA | 797 | 1.0191 |  | Ser | UCU | 582 | 1.7556 |  |
| Ile | AUC | 398 | 0.5091 | trnI-GAU | Thr | ACA | 423 | 1.2944 | trnT-UGU |
| Ile | AUU | 1151 | 1.4718 |  | Thr | ACC | 193 | 0.5908 | trnT-GGU |
| Lys | AAA | 1221 | 1.5992 | trnK-UUU | Thr | ACG | 128 | 0.3916 |  |
| Lys | AAG | 306 | 0.4008 |  | Thr | ACU | 563 | 1.7232 |  |
| Leu | CUA | 387 | 0.8538 | trnL-UAG | Val | GUA | 512 | 1.5376 | trnV-UAC |
| Leu | CUC | 147 | 0.3246 |  | Val | GUC | 131 | 0.3932 | trnV-GAC |
| Leu | CUG | 149 | 0.3288 |  | Val | GUG | 162 | 0.4864 |  |
| Leu | CUU | 580 | 1.2798 |  | Val | GUU | 527 | 1.5824 |  |
| Leu | UUA | 917 | 2.0238 | trnL-UAA | Trp | UGG | 449 | 1 | trnW-CCA |
| Leu | UUG | 539 | 1.1892 | trnL-CAA | Tyr | UAC | 155 | 0.3182 | trnY-GUA |
| Met | AUG | 594 | 2.985 | trnI-CAU | Tyr | UAU | 819 | 1.6818 |  |
