# Supplementary material for: Chloroplast genome sequence of Chongming lima bean (Phaseolus lunatus L.) and comparative analyses with other legume chloroplast genomes
Source: BMC Genomics. 2021 Mar 18;22:194. doi: 10.1186/s12864-021-07467-8 (PMC7977240; doi:10.1186/s12864-021-07467-8)
Supplement: Supplementary file 3 — Additional file 3: Table S3. Repeated sequences of the P. lunatus chloroplast genome. [file 12864_2021_7467_MOESM3_ESM.docx]

Table S3. Repeated sequences of the *P. lunatus* chloroplast genome.

| ID | Repeat I Start | Repeat II Start | Type | Size(bp) | E-Value | Gene | Region |
| --- | --- | --- | --- | --- | --- | --- | --- |
| 1 | 83019 | 84967 | F | 287 | 1.04E-163 | IGS; ycf2 | IRb |
| 2 | 83019 | 145866 | P | 287 | 1.04E-163 | IGS; ycf2 | IRb; IRa |
| 3 | 84967 | 147814 | P | 287 | 1.04E-163 | ycf2; IGS | IRb; IRa |
| 4 | 145866 | 147814 | F | 287 | 1.04E-163 | ycf2; IGS | IRa |
| 5 | 27592 | 27592 | P | 56 | 1.71E-20 | IGS | LSC |
| 6 | 67728 | 67728 | P | 50 | 5.05E-21 | IGS | LSC |
| 7 | 1800 | 115911 | P | 50 | 2.67E-15 | rpl16; ndhA | LSC; SSC |
| 8 | 60944 | 60944 | P | 45 | 1.98E-12 | IGS | LSC |
| 9 | 1798 | 95500 | P | 42 | 2.57E-12 | rpl16; IGS | LSC; IRb |
| 10 | 1798 | 135578 | F | 42 | 2.57E-12 | rpl16; IGS | LSC; IRa |
| 11 | 80518 | 80534 | F | 41 | 3.81E-10 | IGS | IRb |
| 12 | 80518 | 150545 | P | 41 | 3.81E-10 | IGS | IRb; IRa |
| 13 | 80534 | 150561 | P | 41 | 3.81E-10 | IGS | IRb; IRa |
| 14 | 150545 | 150561 | F | 41 | 3.81E-10 | IGS | IRa |
| 15 | 21159 | 21159 | P | 40 | 5.30E-15 | IGS | LSC |
| 16 | 1801 | 19883 | F | 37 | 7.11E-08 | rpl16; ycf3 | LSC |
| 17 | 19883 | 115923 | P | 37 | 7.11E-08 | ycf3; ndhA | LSC; SSC |
| 18 | 31627 | 31676 | P | 36 | 1.46E-10 | IGS | LSC |
| 19 | 1815 | 115911 | P | 35 | 2.90E-08 | rpl16; ndhA | LSC; SSC |
| 20 | 69212 | 69228 | F | 35 | 2.90E-08 | IGS | LSC |
| 21 | 63947 | 63947 | P | 34 | 1.10E-07 | psbL; psbL | LSC |
| 22 | 22738 | 24962 | F | 34 | 3.51E-06 | psaA; psaB | LSC |
| 23 | 28550 | 28599 | P | 33 | 8.68E-11 | IGS | LSC |
| 24 | 91187 | 91187 | R | 33 | 8.68E-11 | IGS | IRb; IRb |
| 25 | 139900 | 139900 | R | 33 | 8.68E-11 | IGS | IRa |
| 26 | 91187 | 139900 | C | 33 | 8.68E-11 | IGS | IRb; IRa |
| 27 | 1808 | 95500 | P | 32 | 3.33E-08 | rpl16; IGS | LSC; IRb |
| 28 | 1808 | 135588 | F | 32 | 3.33E-08 | rpl16; IGS | LSC; IRa |
| 29 | 95500 | 115921 | F | 32 | 4.65E-05 | IGS;ndhA | IRb; SSC |
| 30 | 115921 | 135588 | P | 32 | 4.65E-05 | ndhA; IGS | SSC; IRa |
| 31 | 18783 | 55770 | P | 31 | 5.81E-06 | trnS-GGA; trnS-GCU | LSC |
| 32 | 17661 | 32162 | P | 31 | 1.69E-04 | trnT-UGU; trnT-GGU | LSC |
| 33 | 28496 | 55772 | F | 31 | 1.69E-04 | trnS-TGA; trnS-GCU | LSC |
| 34 | 49407 | 119597 | P | 31 | 1.69E-04 | IGS | LSC; SSC |
| 35 | 56759 | 56776 | P | 31 | 1.69E-04 | IGS | LSC |
| 36 | 106919 | 106919 | P | 31 | 1.69E-04 | ndhF; ndhF | SSC; SSC |
| 37 | 114690 | 114715 | P | 31 | 1.69E-04 | IGS | SSC |
| 38 | 99219 | 99252 | P | 30 | 5.00E-07 | IGS | IRb |
| 39 | 99219 | 131838 | F | 30 | 5.00E-07 | IGS | IRb; IRa |
| 40 | 99252 | 131871 | F | 30 | 5.00E-07 | IGS | IRb; IRa |
| 41 | 131838 | 131871 | P | 30 | 5.00E-07 | IGS | IRa |
| 42 | 80529 | 80545 | F | 30 | 2.17E-05 | IGS | IRb |
| 43 | 80529 | 150545 | P | 30 | 2.17E-05 | IGS | IRb; IRa |
| 44 | 80545 | 150561 | P | 30 | 2.17E-05 | IGS | IRb; IRa |
| 45 | 6597 | 6601 | P | 30 | 6.09E-04 | trnK-UUU; trnK-UUU | LSC |
| 46 | 21161 | 49410 | P | 30 | 6.09E-04 | IGS | LSC |
| 47 | 21167 | 49410 | F | 30 | 6.09E-04 | IGS | LSC |
| 48 | 49408 | 49410 | P | 30 | 6.09E-04 | IGS | LSC |
| 49 | 49420 | 91181 | F | 30 | 6.09E-04 | IGS | LSC; IRb |
| 50 | 49420 | 139909 | P | 30 | 6.09E-04 | IGS | LSC; IRa |
| 51 | 66104 | 66121 | F | 30 | 6.09E-04 | IGS | LSC |
| 52 | 88490 | 88508 | F | 30 | 6.09E-04 | ycf2; ycf2 | IRb |
| 53 | 88490 | 142582 | P | 30 | 6.09E-04 | ycf2; ycf2 | IRb; IRa |
| 54 | 88508 | 142600 | P | 30 | 6.09E-04 | ycf2; ycf2 | IRb; IRa |
| 55 | 142581 | 142599 | F | 30 | 6.09E-04 | ycf2; ycf2 | IRa |
| 56 | 49421 | 91185 | R | 30 | 6.09E-04 | IGS | LSC; IRb |
| 57 | 91181 | 91186 | R | 30 | 6.09E-04 | IGS | IRb |
| 58 | 114706 | 114707 | R | 30 | 6.09E-04 | IGS | SSC |
| 59 | 139904 | 139909 | R | 30 | 6.09E-04 | IGS | IRa |
| 60 | 49421 | 139905 | C | 30 | 6.09E-04 | IGS | LSC; IRa |
| 61 | 91181 | 139904 | C | 30 | 6.09E-04 | IGS | IRb; IRa |

F=forward, P=palindromic, R=reverse, C=complement
