# Supplementary material for: Chloroplast genome sequence of Chongming lima bean (Phaseolus lunatus L.) and comparative analyses with other legume chloroplast genomes
Source: BMC Genomics. 2021 Mar 18;22:194. doi: 10.1186/s12864-021-07467-8 (PMC7977240; doi:10.1186/s12864-021-07467-8)
Supplement: Supplementary file 4 — Additional file 4: Table S4. Simple sequence repeats (SSRs) in the P. lunatus chloroplast genome. [file 12864_2021_7467_MOESM4_ESM.docx]

Table S4. Simple sequence repeats (SSRs) in the *P. lunatus* chloroplast genome.

| Unit | Length | Count | Location | Region | Unit | Length | Count | Location | Region |
| --- | --- | --- | --- | --- | --- | --- | --- | --- | --- |
| A | 8 | 1 | rpl16(intron) | LSC | T | 9 | 1 | petD  (intron) | LSC |
|  | 8 | 1 | IGS | LSC |  | 9 | 1 | rps19 | IRb |
|  | 8 | 3 | trnK-UUU(intron)/matK | LSC |  | 9 | 2 | ndhF | SSC |
|  | 8 | 4 | IGS | LSC |  | 9 | 1 | ccsA | SSC |
|  | 8 | 2 | ycf3(intron) | LSC |  | 9 | 1 | IGS | SSC |
|  | 8 | 5 | IGS | LSC |  | 9 | 6 | ycf1 | SSC |
|  | 8 | 1 | rpoB | LSC |  | 10 | 1 | trnK-UUU  (intron) | LSC |
|  | 8 | 2 | rpoC1(intron) | LSC |  | 10 | 1 | IGS | LSC |
|  | 8 | 2 | rpoC2 | LSC |  | 10 | 1 | IGS | IRb |
|  | 8 | 2 | IGS | LSC |  | 10 | 2 | ycf1 | SSC |
|  | 8 | 1 | ycf4 | LSC |  | 11 | 3 | IGS | LSC |
|  | 8 | 1 | IGS | LSC |  | 11 | 1 | IGS | SSC |
|  | 8 | 1 | clpP(intron) | LSC |  | 11 | 2 | ycf1 | SSC |
|  | 8 | 2 | ycf2 | IRb |  | 11 | 1 | IGS | IRa |
|  | 8 | 4 | ndhF | SSC |  | 12 | 1 | ycf2 | IRa |
|  | 8 | 1 | IGS | SSC |  | 14 | 1 | trnK-UUU  (intron) | LSC |
|  | 8 | 1 | rpl32 | SSC |  | 14 | 1 | IGS | IRb |
|  | 8 | 2 | IGS | SSC |  | 18 | 1 | IGS | LSC |
|  | 8 | 1 | ndhA(intron) | SSC | AT | 10 | 3 | IGS | LSC |
|  | 8 | 1 | IGS | SSC |  | 12 | 1 | IGS | LSC |
|  | 8 | 1 | IGS | IRa |  | 14 | 1 | IGS | LSC |
|  | 9 | 1 | IGS | LSC |  | 16 | 1 | IGS | LSC |
|  | 9 | 1 | trnK-UUU(intron)/matK | LSC | TA | 10 | 4 | IGS | LSC |
|  | 9 | 8 | IGS | LSC |  | 10 | 1 | IGS | SSC |
|  | 9 | 2 | rpoC2 | LSC |  | 12 | 1 | trnK-UUU  (intron) | LSC |
|  | 9 | 3 | IGS | LSC |  | 12 | 1 | clpP(intron) | LSC |
|  | 9 | 1 | petA | LSC | TA | 12 | 2 | IGS | SSC |
|  | 9 | 1 | IGS | LSC |  | 14 | 3 | IGS | LSC |
|  | 9 | 1 | rpl133 | LSC | AAC | 9 | 1 | rpl14 | LSC |
|  | 9 | 2 | IGS | LSC | AAG | 9 | 1 | trnK-UUU  (intron)/matK | LSC |
|  | 9 | 1 | ndhF | SSC |  | 9 | 1 | IGS | LSC |
|  | 9 | 1 | IGS | SSC | AAT | 9 | 1 | rbcL | LSC |
|  | 9 | 1 | ndhA | SSC |  | 9 | 1 | IGS | LSC |
|  | 9 | 1 | ycf1 | SSC | ACC | 9 | 1 | IGS | LSC |
|  | 9 | 1 | rps19 | IRa | ACG | 9 | 1 | psaA | LSC |
|  | 10 | 1 | atpB | LSC | AGA | 9 | 1 | psaA | LSC |
|  | 10 | 1 | trnV-UAC(intron) | LSC |  | 9 | 1 | psaB | LSC |
|  | 10 | 2 | IGS | LSC |  | 9 | 1 | rps14 | LSC |
|  | 10 | 1 | ycf3(intron) | LSC |  | 9 | 1 | IGS | LSC |
|  | 10 | 1 | rpoB | LSC | AGC | 9 | 1 | rpoC1 | LSC |
|  | 10 | 1 | rps2 | LSC | ATA | 9 | 1 | rpoC2 | LSC |
|  | 10 | 1 | trnG-UCC(intron) | LSC |  | 9 | 1 | rps2 | LSC |
|  | 10 | 1 | IGS | LSC | ATG | 9 | 1 | IGS | LSC |
|  | 10 | 1 | cemA | LSC | ATT | 9 | 1 | atpI | LSC |
|  | 10 | 1 | rps18 | LSC | CAG | 9 | 1 | accD | LSC |
|  | 10 | 1 | IGS | SSC | CTG | 9 | 1 | IGS | LSC |
|  | 10 | 1 | IGS | IRa |  | 9 | 1 | ycf4 | LSC |
|  | 11 | 2 | rpoC2 | LSC | CTT | 9 | 1 | ycf4 | LSC |
|  | 11 | 1 | IGS | IRb |  | 9 | 1 | petA | LSC |
|  | 12 | 1 | clpP(intron) | LSC | GAA | 9 | 2 | IGS | LSC |
|  | 12 | 1 | ycf2 | IRb |  | 9 | 1 | rps18 | LSC |
|  | 13 | 1 | IGS | LSC | GGT | 9 | 1 | clpP | LSC |
|  | 13 | 1 | IGS | SSC |  | 9 | 1 | rpoA | LSC |
|  | 14 | 1 | rpl16(intron) | LSC |  | 9 | 1 | rpl2 | IRb |
|  | 14 | 1 | IGS | LSC | TAA | 9 | 1 | rpl2 | IRb |
|  | 14 | 1 | ccsA | SSC |  | 9 | 3 | ycf2 | IRb |
|  | 14 | 1 | IGS | IRa |  | 9 | 2 | ndhB | IRb |
| C | 8 | 1 | ycf2 | IRa | TAC | 9 | 1 | IGS | IRb |
|  | 9 | 1 | psbC | LSC | TAT | 9 | 1 | trnI-GAU  (intron) | IRb |
| G | 8 | 1 | ycf2 | IRb |  | 9 | 1 | rrn23 | IRb |
|  | 8 | 1 | ycf1 | SSC |  | 9 | 1 | ndhF | SSC |
|  | 9 | 1 | IGS | LSC |  | 9 | 1 | IGS | SSC |
| T | 8 | 2 | trnK-UUU(intron) | LSC |  | 9 | 1 | ccsA | SSC |
|  | 8 | 1 | IGS | LSC | TCA | 9 | 1 | IGS | SSC |
|  | 8 | 1 | ycf3(intron) | LSC | TCG | 9 | 1 | ndhA | SSC |
|  | 8 | 11 | IGS | LSC | TCT | 9 | 1 | ndhH | SSC |
|  | 8 | 1 | rpoC1 | LSC |  | 9 | 2 | ycf1 | SSC |
|  | 8 | 1 | rpoC2 | LSC |  | 9 | 1 | rrn23 | IRa |
|  | 8 | 1 | IGS | LSC |  | 9 | 1 | trnI-GAU  (intron) | IRa |
|  | 8 | 1 | atpF | LSC |  | 9 | 1 | IGS | IRa |
|  | 8 | 1 | atpF(intron) | LSC | TGC | 9 | 1 | ndhB | IRa |
|  | 8 | 5 | IGS | LSC | TTA | 9 | 1 | ndhB | IRa |
|  | 8 | 1 | psbT | LSC |  | 9 | 1 | ycf2 | IRa |
|  | 8 | 1 | petB(intron) | LSC | TTC | 9 | 2 | ycf2 | IRa |
|  | 8 | 1 | petD(intron) | LSC |  | 9 | 2 | rpl2 | IRa |
|  | 8 | 3 | IGS | IRb | AAAT | 12 | 1 | ycf1 | IRa |
|  | 8 | 1 | ccsA | SSC | ATAA | 12 | 1 | ycf3(intron) | LSC |
|  | 8 | 1 | ndhD | SSC | ATCT | 12 | 1 | IGS | LSC |
|  | 8 | 1 | IGS | SSC | CTAT | 12 | 1 | IGS | LSC |
|  | 8 | 1 | rps15 | SSC | CTTT | 12 | 1 | IGS | LSC |
|  | 8 | 1 | IGS | SSC | TATC | 12 | 1 | IGS | LSC |
|  | 8 | 7 | ycf1 | SSC | TATT | 12 | 1 | IGS | IRb |
|  | 8 | 2 | ycf2 | IRa | TTGA | 12 | 1 | ndhE | SSC |
|  | 9 | 1 | IGS | LSC | (A)13gaata(AT)5 | 28 | 1 | rpl16(intron) | LSC |
|  | 9 | 1 | trnK-UUU(intron)/matK | LSC | (A)9ga(T)9 | 20 | 1 | IGS | LSC |
|  | 9 | 1 | IGS | LSC | (T)8agattctaa(T)8 | 25 | 1 | rpoC1(intron) | LSC |
|  | 9 | 1 | ycf3(intron) | LSC | (A)9gaacc(T)8 | 22 | 1 | rpoC1 | LSC |
|  | 9 | 5 | IGS | LSC | (A)9gcaagtctc(T)9 | 27 | 1 | IGS | LSC |
|  | 9 | 1 | trnG-UCC(intron) | LSC | (A)8(T)9 | 17 | 1 | IGS | LSC |
|  | 9 | 2 | IGS | LSC | (CAG)3gtatagt(A)8 | 24 | 1 | petA | LSC |
|  | 9 | 1 | accD | LSC | (A)12gaaaata(T)9 | 28 | 1 | IGS | SSC |
|  | 9 | 1 | ycf4 | LSC | (T)10aac(A)9 | 22 | 1 | ycf1 | SSC |
|  | 9 | 1 | clpP(intron) | LSC | (AT)5(T)8 | 17 | 1 | IGS | LSC |
|  | 9 | 2 | IGS | LSC | (TCTA)3tatatat(TCTA)3(TA)6 | 41 | 1 | ndhA(intron) | SSC |
|  | 9 | 1 | petB(intron) | LSC | (T)8[8]3 | 15 | 1 | ycf1 | SSC |
|  | 9 | 1 | IGS | LSC |  |  |  |  |  |
